# Shear Bond Strength of Orthodontic Brackets Fixed with Remineralizing Adhesive Systems after Simulating One Year of Orthodontic Treatment

**DOI:** 10.1155/2015/903451

**Published:** 2015-08-26

**Authors:** Gisele Lima Bezerra, Carlos Rocha Gomes Torres, Mateus Rodrigues Tonetto, Alvaro Henrique Borges, Milton Carlos Kuga, Matheus Coelho Bandeca, Leily Macedo Firoozmand

**Affiliations:** ^1^CEUMA University, Josué Montello 01, Renascença II, 65.075-120 São Luís, MA, Brazil; ^2^São Paulo State University, Avenida Engenheiro Francisco José Longo 777, 12245-000 São José dos Campos, SP, Brazil; ^3^University of Cuiaba, Beira Rio 3100, Jardim Europa, 78065-900 Cuiabá, MT, Brazil; ^4^São Paulo State University, Rua Humaita 1680, 14803-901 Araraquara, SP, Brazil; ^5^Federal University of Maranhão, Avenida dos Portugueses 1966, 65080-805 São Luis, MA, Brazil

## Abstract

The objective of this study is to assess, *in vitro*, the shear bond strength of orthodontic brackets fixed with remineralizing adhesive systems submitted to thermomechanical cycling, simulating one year of orthodontic treatment. Sixty-four bovine incisor teeth were randomly divided into 4 experimental groups (*n* = 16): *XT*: Transbond XT, *QC*: Quick Cure, *OL*: Ortholite Color, and *SEP*: Transbond Plus Self-Etching Primer. The samples were submitted to thermomechanical cycling simulating one year of orthodontic treatment. Shear bond strength tests were carried out using a universal testing machine with a load cell of 50 KgF at 0.5 mm/minute. The samples were examined with a stereomicroscope and a scanning electron microscope (SEM) in order to analyze enamel surface and Adhesive Remnant Index (ARI). Kruskal-Wallis and Mann-Whitney (with Bonferroni correction) tests showed a significant difference between the studied groups (*p* < 0.05). Groups XT, QC, and SEP presented the highest values of adhesive resistance and no statistical differences were found between them. The highest frequency of failures between enamel and adhesive was observed in groups XT, QC, and OL. Quick Cure (QC) remineralizing adhesive system presented average adhesive resistance values similar to conventional (XT) and self-etching (SEP) adhesives, while remineralizing system (OL) provided the lowest values of adhesive resistance.

## 1. Introduction

Orthodontic practice is in constant improvement, enabling the use of new techniques and materials benefiting both patient and professional. Thus, attempts to inhibit the development of carious lesions in patients under orthodontic treatment have been focused on the control of bacterial biofilms around the orthodontic accessories [1, 2].

Orthodontic braces physically alter the microbiological environment leading to an increase in the formation of bacterial biofilm due to the formation of a higher number of biofilm retention sites [3]. An increase in the incidence of initial carious lesions and inflammation of the gum tissue is verified in patients submitted to orthodontic treatment with fixed braces [4].

Some studies [5, 6] have been investigating materials that can be used as an alternative to adhesive systems and composite resins, aiming to prevent enamel demineralization around the orthodontic brackets.

In Orthodontics, the adhesive system maintains the orthodontic accessories in accurate places during the entire treatment, helping in the reestablishment of an ideal occlusion. The orthodontic treatment must correct the occlusion in a satisfying way; however, it must not alter the preexisting health of teeth and supporting tissues; otherwise, the treatment benefits might be questioned.

The current tendency is the improvement of adhesive systems with simplified application protocol altogether with promoting satisfying adhesive resistance, reducing procedure errors, and minimizing damage to tooth structure [7]. Self-etching systems that have acidic components reduce the inconveniences of excessive demineralization of the structure of the tooth and provide a decrease in the number of surgical procedures [8, 9]. Adhesive systems that allegedly have remineralizing properties are displayed in the market [5, 6]; however, the longevity of the treatment was not confirmed in the literature.

Thus, the biomechanical behavior of these new systems has to be investigated so that they can be effectively applied during the entire orthodontic treatment. As a major part of the studies evaluates the adhesive resistance of orthodontic brackets just right after their installation [6, 8, 10, 11], a long time treatment evaluation is an important factor.

Considering these questions regarding different adhesive systems and the lack of studies assessing the adhesive resistance of remineralizing adhesives, the present study aims to perform the shear bond strength of orthodontic brackets fixed with remineralizing adhesive systems submitted to thermomechanical cycling, simulating one year of orthodontic treatment. The null hypothesis tested is that there is no statistical difference in the resistance values when conventional, remineralizing, and self-etching systems are applied.

## 2. Materials and Methods

The present experiment used 64 bovine incisors just extracted, cleaned, and stored in distilled water. This research project was approved by Animal Experimentation Ethics Committee (protocol number 073/2013). The criteria of inclusion for tooth selection were the following: tooth enamel with no fissures and no previous application of chemical agents such as hydrogen peroxide, alcohol, and formalin. The sample size was calculated considering *α* equal to 5%, power of Kruskal-Wallis test 75%. The result was a size (*n*) of 16 teeth in each group (PASS 11. NCSS, LLC, Kaysville, Utah, USA).

The bovine teeth were sectioned in the cervical line and the roots, which are disposed. The dental pulp was extirpated with the aid of a dentin curette (Duflex Lucas number 86, SSWhite, Rio de Janeiro, RJ, Brazil) and the pulp chamber was irrigated with distilled water, air-dried, and obliterated with utility wax.

The teeth were positioned in 25 mm × 20 mm PVC cylindrical tubes (Tigre, Joinville, SC, Brazil), keeping the buccal surface positioned at the bottom of the base, and embedded in acrylic resin (VIPI, São Paulo, SP, Brazil). In order to obtain plain buccal surfaces, parallel to the block base, these were submitted to plaining and polishing with sandpaper (granulations 200, 400, 600, and 1200) (3 M, Sumaré, SP, Brazil) with the aid of a polishing machine (Panambra Técnica Imp. Exp. Ltda., São Paulo, SP, Brazil), with irrigation and constant uniform pressure.

The adhesive systems presented in Table 1 were applied in the making of the experimental groups.

### 2.1. Fixation of the Brackets

Sixty-four stainless steel orthodontic brackets were used for upper central incisors with 1.5 mm high and 4.0 mm wide mesh bases (Roth 0.022′′ × 0.030′′, KIRIUM Abzil Ind. & com. Ltda., São José do Rio Preto, SP, Brazil). The buccal surface of the teeth enamels underwent prophylaxis with fluoride-less pumice stone (SSWhite, Rio de Janeiro, RJ, Brazil) and water for ten seconds.

Groups XT, QC, OL, and SEP were submitted to the application of the adhesive systems according to the specifications in Table 2. Maximum pressure was applied in the bracket bonding or the standardization of the strength exercised and the thickness of the resin layer. The excess was removed before polymerization with a dental explorer (Duflex number 5, São Paulo, SP, Brazil). A properly trained and calibrated operator performed all procedures.

The adhesive system and resin underwent photopolymerization with the device fast-curing cordless LED light (3 M ESPE dental, Landsberg am Lech, Germany) with a radiometer-checked (Gnatus, Ribeirão Preto, SP, Brazil) light intensity of 800 mW/cm^2^.

### 2.2. Thermomechanical Cycling

All the testing groups were submitted to thermomechanical variation cycles using a dental wear simulator (ER 11000, ERIOS, São Paulo, SP, Brazil). The specimens underwent thermal cycles between 5 and 55°C with a dwell time of 30 s. During this procedure, a force of 50 N was delivered at 1 Hz. In order to simulate a one-year clinical treatment according to Gale and Darvell [12], 100,000 mechanical cycles and 500 thermal cycles (ISO 11405) were performed.

### 2.3. Shear Bond Strength Test

A universal testing machine (EMIC, São José dos Pinhais, PR, Brazil) with a load cell of 50 Kg was used to perform the shear bonds strength test, concurrently applied to the buccal surface of the enamel in the incisal-cervical direction close to the enamel/adhesive junction at 0.5 mm/min until it fractured. The strength required to remove the accessories was measured in Newton (N) and the shear bond strength in megapascal (MPa). The results were obtained with the aid of the computer software (TESC) connected to the universal testing machine EMIC.

### 2.4. Evaluation under Stereomicroscope and Scanning Electron Microscope (SEM)

After shear testing, the samples were analyzed in a stereoscopic magnifier (Kozo Optical and Electronical Instrumental, Nanjing-Jiangsu, China) with 20x magnification to determine the Adhesive Remnant Index (ARI). This measurement was performed in accordance with the scores varying from 0 to 3. Score 0 = no amount of adhesive material adhered to the tooth; 1 = less than half of the adhesive material adhered to the tooth; 2 = more than half of the adhesive material adhered to the tooth; and 3 = all adhesive material adhered to the tooth, including bracket mesh impression.

For SEM, the samples were dehydrated during 5 h in increasing concentrations of alcohol (70%, 90%, and 99%) and they were placed on metal stubs, labeled and sputter-coated with 120-Å thick gold palladium (MED 020; BAL-TEC, Balzers, Liechtenstein). They were then analyzed under a scanning electron microscope (SEM) operating at 15 KV in order to visualize the adhesive remnant and/or enamel condition after the removal of the brackets. The capture of the images was performed with software coupled to the MEV (Inspect 550, Fei), allowing the obtainment of photomicrographs.

### 2.5. Statistical Analysis

The data were analyzed with Kruskal-Wallis and Mann-Whitney with Bonferroni correction tests to verify the difference the difference between the studied groups, as the data distribution was considered abnormal according to Kolmogorov-Smirnov tests. The significance level of *p* < 0.05 was applied.

The Adhesive Remnant Index data presented as an ordinal qualitative variable were analyzed with Kruskal-Wallis and Dunn tests.

The analyses were performed using the statistics software SPSS Statistics version 20.0 (IBM, Armonk, NY, USA).

## 3. Results

### 3.1. Shear Bond Strength Test

Descriptive and inferential statistics of the adhesive resistance in the studied groups are represented in Table 3.

**Figure 1:**
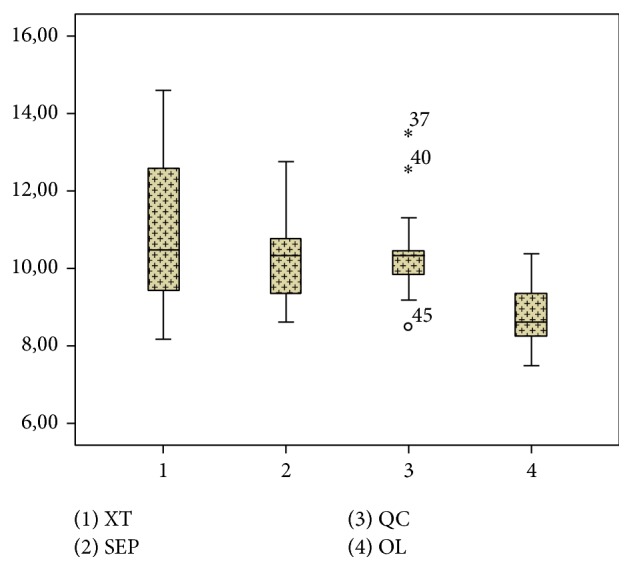
Box-plot, median, and standard deviation of the values of shear bond strength (MPa) in the different adhesive systems and studied groups.

Shear bond strength tests after one-year period simulation demonstrated that the groups XT, SEP, and QC did not present statistical differences between them and provided the highest shear bond strength values when compared with group OL (Figure 1).

### 3.2. Stereomicroscope ARI Evaluation

ARI frequency distribution is represented in Table 4, where it was possible to observe that the score “0” was predominant in the groups XT, QC, and OL, which represents adhesive failure.

The condition of highest adherence, ARI = 3 (all adhesive material adhered to the tooth, including bracket mesh impression), was observed in the groups SEP and OL.

### 3.3. Scanning Electron Microscope (SEM) Analysis

**Figure 2:**
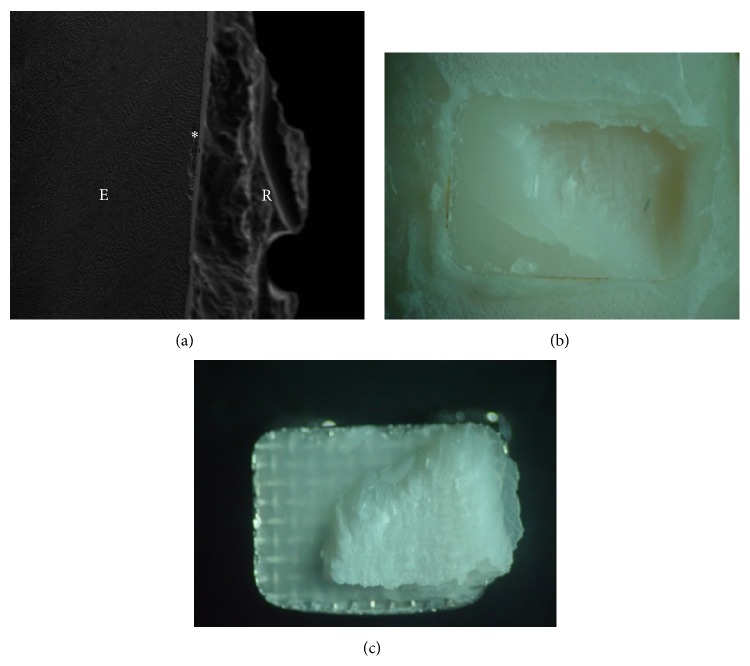
(a) Images of group QC. (a) Magnification of 500x showing injuries in the enamel with the bracket debonding. (b) Images in stereomicroscope of the tooth buccal and (c) bracket base.

The group bonded with the antibacterial adhesive Quick Cure (QC) presented cracks and depression on the enamel surface after removing the brackets, presenting damage to the tooth structure (Figure 2).

**Figure 3:**
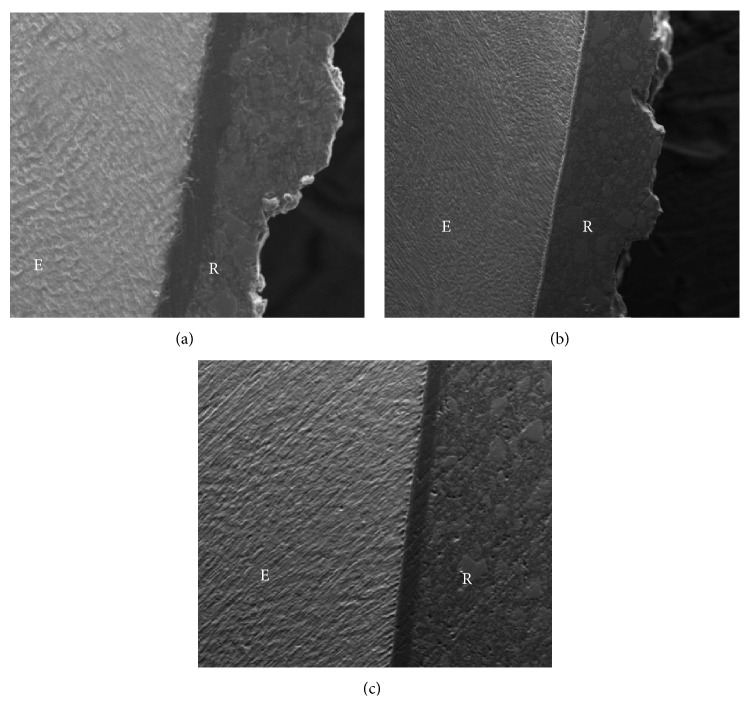
(a) Microimages of group XT. Magnification of 500x. (b) Microimages of group SEP. Magnification of 500x. (c) Microimages of group OL. Magnification of 500x.

In the other groups XT, SEP, and OL, there was no damage to the enamel surface that could be visualized at the SEM (Figure 3).

## 4. Discussion

White spot lesions are usually observed in orthodontic patients, due to cleaning difficulties [13, 14], and long periods involving the treatment. Therefore, the increasing search for the development of materials and techniques aiming to reduce the damaging effects caused by the use of fixed orthodontic braces is observed. After the treatment simulation, the remineralizing adhesive systems presented various results according to the composition/brand of the materials. Thus, the null hypothesis was rejected.

The bonding resistance of orthodontic brackets is usually verified 24 hours after installation [6, 8, 10, 11]. However, both primary stability and longevity of the brackets are extremely important, as the brackets are susceptible to a variety of forces inside the oral cavity. The adhesive resistance is influenced by many factors such as bracket surface area, adhesion technique, type of adhesive applied, bracket base design, and adhesion protocol [15, 16]. Ideally, an orthodontic bracket must reproduce a good orthodontic strength, support masticatory loads, and be easily removable at the end of the treatment, without causing injuries to the tooth surface. Nevertheless, a substantial part of* in vitro* studies [10, 11] does not use any type of artificial fatigue prior to the assessment of adhesive resistance, but thermomechanical cycling is recommended [17] in order to consider its real adhesive longevity.

The assessed adhesive/resin systems presented adhesive resistance values in accordance with the parameters found in the literature [11, 16], with group OL being the only one to present lower values. However, the average values obtained by the adhesive system (OL) were higher to those indicated by Reynolds [18], showing that the bonding resistance must reach a value over 60 Kgf/cm^2^ (5,88 MPa)/80 Kgf/cm^2^ (7,84 MPa) to be properly applied for clinical needs.

After the one-year simulation, the shear bond strength of the self-etching system Transbond Self-Etching (10,3 MPa) and remineralizing system Quick Cure (10,4 MPa) were statistically similar when compared with the conventional system Transbond XT (10,8 MPa), as verified in previous studies [19]. On the other hand, the group Ortholite (8,8 MPa) presented the lowest adhesive resistance average values, indicating that these results seem to be material-dependent; the remineralizing system Quick Cure presented the same shear bond strength as the standard system (Transbond XT); and the remineralizing system (Ortholite) presented lower adhesive resistance, confirming previous studies [20]. The chemical formulation of the Ortholite adhesive presents fluoride compounds, but manufacturers do not specify the components used or their amount. Thus, as seen in the literature [6], some different results are obtained depending on the formulations handled by each manufacturer.

According to the information given by the manufacturer (Table 1), adhesive systems have fluorine in their composition in the form of sodium fluoride, hexafluorophosphate, fluoridric complex, and fluoride composites for Quick Cure, Transbond XT, Transbond Self Etching, and Ortholite Cure, respectively. Therefore, even if remineralizing composites are added to the material, it is necessary to verify its influence on the bonding resistance.

Evaluating concentrations of calcium, phosphorus, silicon, and carbon, Chow et al. (2011) [5] verified that there was no significant statistical difference between the adhesives Transbond XT and Quick Cure. However, regarding anticariogenic potential, Quick Cure presented significantly lower adherence of* S. mutans *and a decrease of 23.6% of the lesion depth area in relation to the control group, whereas Transbond XT presented an increase of 3.2% of the lesion depth area in relation to the control. Considering the toxicity of the materials, Malkoc et al. (2010) [21] indicate that the adhesive system Transbond XT demonstrated a decrease in the number of vital fibroblasts when compared to the adhesive system Quick Cure and the control group.

Studies have been carried out intending to develop materials that prevent demineralization and/or promote remineralization of tooth enamel adjacent to the orthodontic brackets [5, 6]. ACP (amorphous calcium phosphate) is also suggested as a cooperator or independently used as a prevention agent. Nevertheless, some studies [22, 23] demonstrate that systems containing ACP can reduce the bonding strength of bonded brackets in relation to conventional adhesives.

The analysis of the adhesive interface after shear bond strength test indicated a higher frequency of faults in the interface adhesive-enamel, which was also observed in studies* in vivo* [24]. The exception was the group* Transbond Self-Etching* that, according to the literature, can offer potential benefits when compared with systems that promote total acid etch, due to its smaller reversible alterations on the tooth enamel surface [25]. Through the visualization using SEM, it was verified that the antibacterial adhesive Quick Cure, which presented adhesive resistance values higher than those of Ortholight Cure and similar to conventional adhesive (XT) and self-etching adhesive (SEP), presented severe damage to the buccal surface, with fractures in the enamel resulting from the debonding of the brackets. The other groups presented no significant permanent damage to the tooth enamel. Based on this information, further studies are required to elucidate the influence of these materials on the adhesion to the tooth enamel.

## 5. Conclusions

Considering the limitations of this study, after the one-year orthodontic treatment simulation through thermomechanical cycling, it was possible to observe that

1. Quick Cure (QC) adhesive remineralizing system presented average adhesive resistance values such as conventional (XT) and self-etching (SEP) systems;
2. Ortholite Cure (OL) remineralizing system presented lower adhesive resistance values;
3. except the group treated with self-etching adhesive (SEP), most of the faults occurred in the interface enamel-adhesive.

## Figures and Tables

**Table 1:** Experimental groups.

Groups	Adhesive systems/composition	Commercial brand/lot/expiration date
XT	**Transbond XT: light cure adhesive** *Adhesive*: triethylene glycol dimethacrylate (TEGDMA) and BISGMA *Resin*: silica, BISGMA, N-dimethyl benzocaine, and hexafluorophosphate	3M Unitek Orthodontics Products South Peck Road, Monrovia, USA L: 1308100970 Exp.: 06/15

QC	**Quick Cure: fluoride** *Adhesive*: bisphenol dimethacrylate, hydroxyethyl methacrylate, and acetone *Resin*: silica, BISGMA, triethylene-glycol-dimethacrylate (TEGDMA), and sodium fluoride	Reliance Orthodontic products, Inc. Itasca, Illinois, USA L: 123643 Exp.: 05/14

OL	**Ortholite Cure: color change** *Adhesive*: phosphate acrylic monomer, ethanol, acetone, and amine *Resin*: triethylene glycol dimethacrylate (TEGDMA), BISGMA, amine, and fluoride compounds	OrthoSource, USA Sherman way, Hollywood, USA L: CUKA Exp.: 04/14

SEP	**Transbond Plus Self-Etching Primer** *Adhesive*: mono- and di-HEMA phosphates, distilled water, and fluorine compounds	3M Unitek Orthodontics Products South Peck Road, Monrovia, USA L: 488941c Exp.: 03/14

**Table 2:** Application methods of the adhesives systems used in this study.

Groups	Application method
XT, QC, and OL	(i) Etching phosphoric acid 37%, 30 s on the enamel (ii) Washing for 30 s and air jet drying (iii) Active application of 2 layers of the adhesive (5 s) (iv) Using a brief air jet to evaporate the solvent and make sure there is a thin, uniform layer (v) Photopolymerization for 20 s. (vi) Resin application and photopolymerization for 10 s, each surface (mesial, distal, cervical, and incisal).

SEP	(i) Active application of 2 layers of the adhesive (5 s) (ii) Use of a brief air jet (iii) Photopolymerization for 20 s (iv) Resin application and photopolymerization for 10 s in each surface (mesial, distal, cervical, and incisal)

**Table 3:** Shear bond strength (MPa) analysis of orthodontic adhesives.

Groups	Adhesive systems	*N*	Mean (standard deviation)	Median (MPa)	25–75%
XT	Transbond XT	16	10.8 (1.8)^A^	10.4	9.2–12.6
QC	Quick Cure	16	10.4 (1.2)^A^	10.3	9.8–10.5
OL	Ortholight Cure	16	8.8 (0.7)^B^	8.6	8.2–9.4
SEP	Transbond Self-Etching Primer	16	10.3 (1.1)^A^	10.3	9.2–10.7

^*∗*^Different letters indicate statistically significant differences according to post hoc tests with Bonferroni correction (*p* < 0.005).

**Table 4:** Adhesive remnant scores (ARI) of the four groups [*n* (%)], average score, median, and statistical difference found.

Group (*n* = 16)	ARI score				Average score (median)	Dunn
	0	1	2	3		
XT	10 (62.5%)	2 (12.5%)	3 (18.8%)	1 (6.3%)	0.69 (0)	A
QC	7 (43.8%)	2 (12.5%)	4 (25%)	3 (18.8%)	1.19 (1)	ABC
OL	7 (43.8%)	0 (0%)	3 (18.8%)	6 (37.5%)	1.50 (2)	AB
SEP	2 (12.5%)	3 (18.8%)	5 (31.3%)	6 (37.5%)	1.94 (2)	B

^*∗*^Different letters indicate statistically significant differences (*p* < 0.05).
